# Bioprospecting microbes and enzymes for the production of pterocarpans and coumestans

**DOI:** 10.3389/fbioe.2023.1154779

**Published:** 2023-04-28

**Authors:** Fernando Perez Rojo, J. Jane Pillow, Parwinder Kaur

**Affiliations:** ^1^ UWA School of Agriculture and Environment, The University of Western Australia, Perth, WA, Australia; ^2^ UWA School of Human Sciences, The University of Western Australia, Perth, WA, Australia

**Keywords:** microbial bioprospecting, enzyme bioprospecting, microbial-based production, isoflavonoids, pterocarpans and coumestans

## Abstract

The isoflavonoid derivatives, pterocarpans and coumestans, are explored for multiple clinical applications as osteo-regenerative, neuroprotective and anti-cancer agents. The use of plant-based systems to produce isoflavonoid derivatives is limited due to cost, scalability, and sustainability constraints. Microbial cell factories overcome these limitations in which model organisms such as *Saccharomyces cerevisiae* offer an efficient platform to produce isoflavonoids. Bioprospecting microbes and enzymes can provide an array of tools to enhance the production of these molecules. Other microbes that naturally produce isoflavonoids present a novel alternative as production chassis and as a source of novel enzymes. Enzyme bioprospecting allows the complete identification of the pterocarpans and coumestans biosynthetic pathway, and the selection of the best enzymes based on activity and docking parameters. These enzymes consolidate an improved biosynthetic pathway for microbial-based production systems. In this review, we report the state-of-the-art for the production of key pterocarpans and coumestans, describing the enzymes already identified and the current gaps. We report available databases and tools for microbial bioprospecting to select the best production chassis. We propose the use of a holistic and multidisciplinary bioprospecting approach as the first step to identify the biosynthetic gaps, select the best microbial chassis, and increase productivity. We propose the use of microalgal species as microbial cell factories to produce pterocarpans and coumestans. The application of bioprospecting tools provides an exciting field to produce plant compounds such as isoflavonoid derivatives, efficiently and sustainably.

## 1 Introduction

Bioprospecting enables researchers to explore biodiversity, and identify novel molecules, enzymes and microbes relevant to research and industrial applications (Beattie et al., 2011; AfifaHussain et al., 2022). Bioprospecting strategies can be sub-divided into microorganisms (metagenomics), enzymes (transcriptomics/proteomics), or individual molecules (metabolomics). Microbial bioprospecting explores vastly diverse microorganisms, which constitute more than two-third of global life forms, present in a diverse range of environments including extreme ones (Becker and Wittmann, 2020). Metagenomics tools provide information from even non-culturable microbes, being a rich source of novel metabolic pathways, enzymes and their related catalyzed products. The combination of omics-based technologies allows researchers to link genes with enzymatic pathways and metabolite biosynthesis, fuelling the discovery of novel molecules for diverse applications (Bansal et al., 2022). To understand how these molecules are produced, the whole enzymatic pathway needs to be identified, including the genes involved in the synthesis and its regulatory framework. *In silico* enzyme bioprospecting allows the identification of novel homologous sequences, and the possibility to model and trial enzymatic activities. After this *in silico* approach, heterologous biosynthetic pathways can be incorporated into model microorganisms using a combination of the most efficient enzymes through synthetic biology tools. Optimized microbial chassis are proven to increase yield and reduce the production’s environmental impact towards a circular bioeconomy model (Krüger et al., 2020). Bioprospecting strategies for enzymes and chassis offer an unprecedented number of novel biocatalysts for a more efficient and sustainable production.

The use of microbes such as *Saccharomyces cerevisiae* and *Escherichia coli* is one possible strategy to overcome the limitations of plant-based production systems. As the isoflavonoids derivatives pterocarpans and coumestans are mainly produced in plant species, microbes provide an interesting alternative for boosting production titer, sustainably. Additionally, bioprospecting microbes that naturally produce isoflavonoids allow the diversification of production chassis, moving away from model organisms and allowing the implementation of even more sustainable solutions.

Primary metabolites such as amino acids, carbohydrates and lipids are essential for the growth and development of living organism. Secondary metabolites are typically modified chemical derivatives of these metabolites; such modifications include methylation, glycosylation, and hydroxylation (Twaij and Hasan, 2022). Though not essential, secondary metabolites have significant influence in the survivability of organisms and, in the case of plants, are quite often vital for plant-biome interactions.

The secondary metabolites, pterocarpans and coumestans, have clinical potential in both plants and humans. These isoflavonoid derivatives are associated with antimicrobial and antifungal properties (considered a phytoalexin), and its action is explored to deal with plant diseases such as fungal infections (Gupta et al., 2022). Furthermore, they have shown some promise for the treatment diseases of Alzheimer’s disease, osteoporosis, and cancer (Dixit et al., 2015; Li et al., 2021; Tu et al., 2021). Thus, isoflavonoid derivatives merit further exploration with respect to not only its clinical effects but also its method of production.

This review summarizes research efforts to identify the biosynthetic pathways and microbial-based production systems for these plant secondary metabolites, identifying knowledge gaps and providing insights into unexplored production systems. The scope for this review is limited to the most cited coumestans and pterocarpan molecules: medicarpin, pisatin, maackiain, glyceollins, coumestrol, wedelolactone, psoralidin, and glycyrol. The main purpose of this review is to consolidate the link between the current knowledge of pterocarpans and coumestans biosynthesis (including gaps and limitations) and bioprospecting strategies to identify enzymes (address gaps) and novel production systems. We also discuss microbial and enzyme bioprospecting solutions to enhance the sustainable production of pterocarpans and coumestans using microbial-based production systems.

## 2 Isoflavonoids: A key family of phenolics

Phenolics are vastly diverse molecules distributed within several plant species (Tohge et al., 2013). They contain an aromatic ring and at least one hydroxyl group, and are derivatives of the aromatic amino acids, phenylalanine and tyrosine, through the shikimate or acetate pathways (Bravo, 1998). They are related to pigmentation and astringency, but also have diverse roles in plant defense against pathogens (Durazzo et al., 2019). The phenolics biosynthetic pathway starts from the amino acids phenylalanine and tyrosine, from which the enzymes phenylalanine ammonia-lyase (PAL) and tyrosine ammonia-lyase (TAL) generate cinnamate. Subsequently, a subfamily of 4-coumarate ligases produces different CoA-esters, including p-Coumaroyl-CoA, a key member of polyphenols biosynthetic pathways (Vogt, 2010). The phenolic family comprises phenolic acids, tannins, stilbenes, lignans, and flavonoids. Multiple applications are investigated for all these groups from the pharmaceutical, food, and cosmetic industries (Albuquerque et al., 2021).

Flavonoids are the largest family of polyphenols, ubiquitous in land plants and some algal species, potentially sharing a pathway from a common ancestor (Yonekura-Sakakibara et al., 2019). Epidemiological and meta-analysis studies show flavonoids exert positive human health effects with antioxidant, hepatoprotective, antibacterial, anti-inflammatory, antiviral, and anti-cancer properties (Kumar and Pandey, 2013; Amawi et al., 2017; Jiang et al., 2019). The substitution of chemical groups in the flavonoid backbone is related to its biological and chemical properties (Teng and Chen, 2019). Further chemical modifications such as O- and C-methylation and O- and C-glycosylation alter pharmacokinetic variables such as solubility and chemical stability, shifting the molecule’s bioavailability (Sajid et al., 2021a). Seven subclasses are categorized according to the degree of oxidation: flavanones, flavonols, flavones, flavanols, chalcones, anthocyanidins, and isoflavonoids.

**FIGURE 1:**
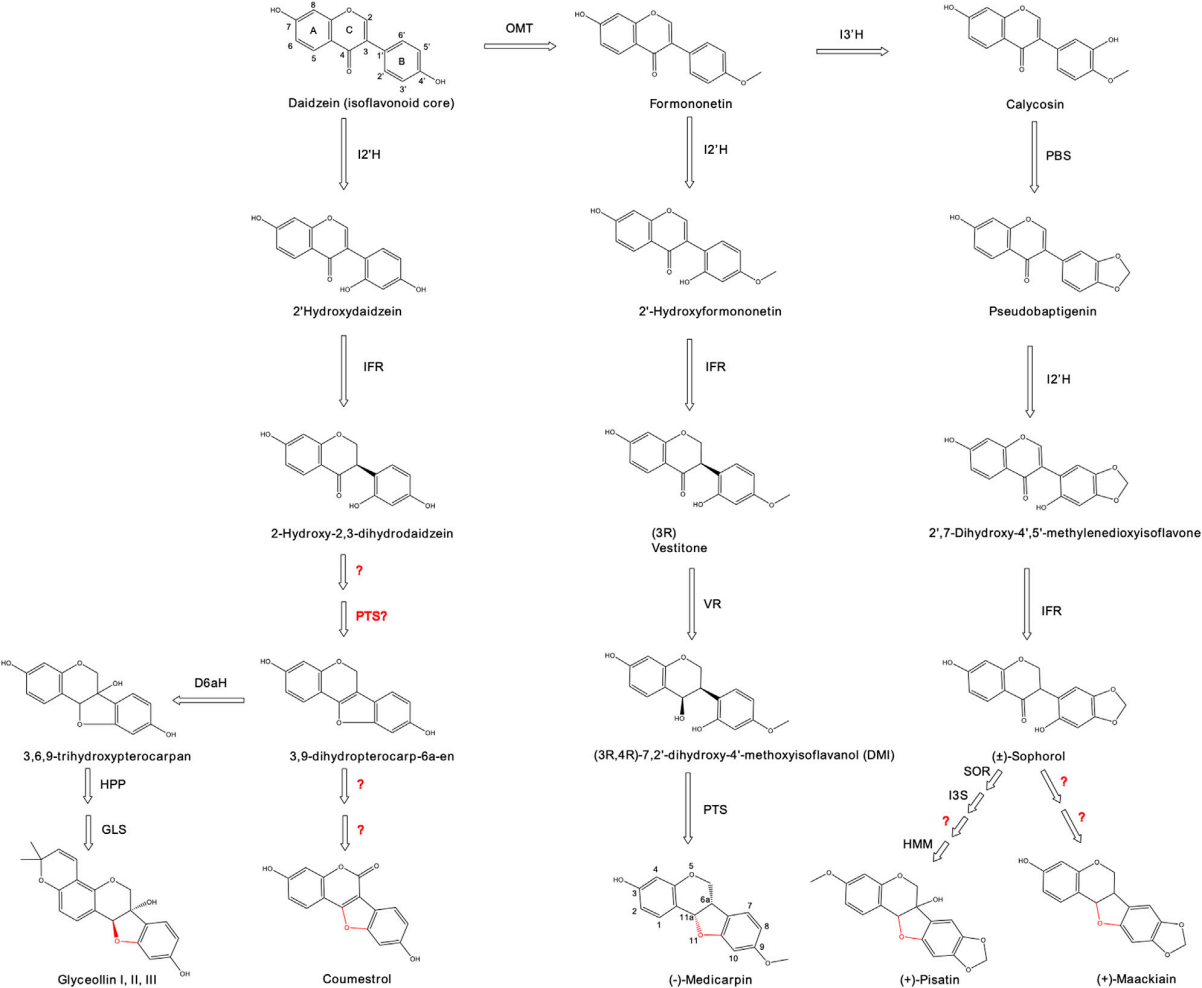
Biosynthetic pathway for the production of coumestans and pterocarpans from the precursor, daidzein. Numbers for identifying carbon positions are shown for daidzein (isoflavonoid core) and medicarpin (carbon numbering varies with the formation of the fourth ring). The formation of a fourth ring between carbons 6 and 11 is highlighted in red. Abbreviations: OMT = isoflavanone 4′-O-methyltransferase; I3′H = isoflavone 3′-hydroxylase; I2′H = isoflavone 2′-hydroxylase; IFR = isoflavone reductase; PBS = pseudobaptigenin synthase; VR = Vestitone reductase; PTS = pterocarpan synthase; SOR = sophorol reductase; I3S = isoflav-3-ene synthase; HMM = (+)-6a-hydroxymaackiain 3-O-methyltransferase; DHPH = 3,9-dihydroxypterocarpan 6a-hydroxylase; GLS = glyceollin synthase; HPP = trihydroxypterocarpan prenyltransferase; D6aH = 3,9-dihydroxypterocarpan 6a-hydroxylase. Question marks indicates unknown enzymatic reactions.

Isoflavonoids main structure is composed of three rings, two benzene rings (A and B) linked by a pyran ring (C). Isoflavonoids is the only subclass of flavonoids with the B ring in position 3 instead of position 2 (Figure 1). More than 2,400 isoflavonoids are reported, of which isoflavones are the most extensively cited group, predominantly found in legume species such as soy, alfalfa and chickpea (Dixon, 1999). During biosynthesis of isoflavonoids, p-Coumaroyl-CoA is converted to a chalcone product by chalcone synthase (CHS), and is then catalyzed into the precursors naringenin and liquiritigenin by the chalcone isomerase (CHI). From these precursors, the isoflavonoids, genistein and daidzein, are generated due to the action of the isoflavone synthase (IFS) (Nabavi et al., 2020). Genistein and daidzein, along with the methylated biochanin A and formononetin, are widely described with multiple osteogenic, anticancer and antioxidant properties (Brodowska, 2017; Sajid et al., 2021b). A large range of isoflavonoid derivatives is produced from these molecules, including pterocarpans and coumestans.

## 3 Pterocarpans and coumestans: Multipurpose molecules with incomplete biosynthetic pathways

Pterocarpans and coumestans are derivatives from the isoflavonoid daidzein. They share the differential feature of a formation of a fourth ring between C and B rings, presenting two asymmetric carbons at positions C-6a and C-11a. The chemical structure of relevant pterocarpans and coumestans is reported in Figure 1.

### 3.1 Production of pterocarpans

Pterocarpans are an extensive group of more than 400 isoflavonoid derivatives with described beneficial human health effects (Jiménez-González et al., 2008; Goel et al., 2013). There are two possible configurations, levorotatory (−) pterocarpans (configuration 6aR, 11aR) and dextrorotatory (+) pterocarpans with the opposite configuration. Within this classification, multiple legume species produce (−) pterocarpans, while just a few plant species, such as peanut (*Arachis hypogaea*), produce (+) pterocarpans (Strange et al., 1985).

Medicarpin is a pterocarpan widely cited due to its diverse range of applications. It is primarily found in legume species, being the main isoflavone in alfalfa (*Medicago sativa L.*) (Du et al., 2010). Medicarpin chemical structure is composed of a pterocarpan core with a hydroxyl group on position C-3 and a methyl group on position C-9. Besides its function as a natural antioxidant, medicarpin is a promising neuroprotective agent for Alzheimer’s disease, and also as a bone regeneration agent to treat osteoporosis (Dixit et al., 2015; Li et al., 2021; Kim et al., 2022). Medicarpin also has potential uses in the agricultural sector. As it is a phytoalexin with antimicrobial effects, medicarpin confers powdery mildew resistance to alfalfa species (Gupta et al., 2022). The first enzyme involved in the biosynthesis of medicarpin is the isoflavanone 4′-O-methyltransferase (OMT). It is a single-step reaction that incorporates a methyl group on the C- 4′ (ring B) of the precursor daidzein, resulting in formononetin as the product. The next step is the formation of 2′-hydroxyformononetin by the enzyme isoflavone 2′ hydroxylase (I2′H), which performs an oxidation at the C-6′ position (ring B). This enzyme belongs to the cytochrome P450 superfamily (class CYP81E1/E7) and produces a set of 2′ hydroxyflavones using different acceptor molecules. It was identified in chickpea, alfalfa and licorice species (Hinderer et al., 1987; Akashi et al., 1998; Liu et al., 2003). The following step in medicarpin synthesis involves the enzyme isoflavone reductase (IFR) that reduces 2′-hydroxyformononetin to the (3R)-2′-hydroxyisoflavanone, reported also as (3R)-vestitone (Paiva et al., 1991). This enzyme is particularly interesting because it introduces a chiral centre using an achiral precursor. IFR was first reported in alfalfa, but homologous sequences were identified from several legume species (Cheng et al., 2015). Vestitone reductase (VR) is the next enzymatic step, which reduces the keto group from position C-4 (ring C) creating the alcohol derivative 7, 2′-dihydroxy4′-methoxyisoflavanol (DMI). VR was first described in *M. sativa* and lately in the pea *Pisum sativum* (Guo et al., 1994a; DiCenzo and VanEtten, 2006). The final step utilizes the enzyme pterocarpan synthase (PTS) to create the characteristic fourth ring between positions C-6a and C-11a, using the two hydroxyl residues as substrates (Fischer et al., 1990). This reaction, which produces (−)-medicarpin from DMI, was described in alfalfa (Guo et al., 1994b). PTS is a dirigent, or stereochemistry-altering, enzyme that also catalyzes the formation of (+)-medicarpin, where the configuration of the hydrogen attached to the C-3 position determines which enantiomer (+ or −) will be produced.

Pisatin and maackiain are phytoalexins from the pterocarpans family that present multiple clinical effects. Maackiain was demonstrated as a potent anti-oxidant and anti-inflammatory agent with anti-sepsis, anti-allergies and neuroprotective actions, among others (Mizuguchi et al., 2015; Tsai et al., 2020; Bai et al., 2022). Besides its antimicrobial activities, pisatin has not been extensibly explored for clinical applications. Pisatin was the first (+)-pterocarpan identified, coming from the pea *P. sativum*, and maackiain was isolated from the Chinese medicinal herb *Sophora flavescens* (Cruickshank and Perrin, 1960; He et al., 2015)*.* The enzyme isoflavone 3′-hydroxylase (I3′H) adds a hydroxyl group at the position C-3′ of the precursor formononetin, as the first step for both pisatin and maackiain biosynthesis. The metabolite produced, named calycosin, is then converted into pseudobaptigenin by the enzyme pseudobaptigenin synthase (PBS) (Liu et al., 2003). The pathway continues with the already mentioned I2′H and IFR enzymes, which introduces chirality to obtain the precursor compounds (+)-sophorol and (−)-sophorol, of different enantiomers. From there, the biosynthetic steps for the synthesis of maackiain are not fully identified yet, but it is believed that downstream enzymes from (+)-pisatin may be involved (DiCenzo and VanEtten, 2006). The production of (+)-pisatin involves the enzyme sophorol reductase (SOR) that produces (3R and 4R)-7, 2′-dihydroxy-4′5′-methylenedioxyisoflavanol (DiCenzo and VanEtten, 2006). The next step is catalyzed by isoflav-3-ene synthase (I3S) that converts the precursor into 7, 2′-dihydroxy-4′, 5′-methylenedioxyisoflav-3-ene (DMDIF). The subsequent enzymatic reaction is still not identified, but it produces the molecule (+)-6a-hydroxymaackiain using DMDIF as a precursor. The final step requires the action of the enzyme (+)-6a-hydroxymaackiain 3-O-methyltransferase (HMM), which adds a methyl group at the C-3 position to generate the final metabolite, (+)-pisatin (Wu et al., 1997).

Glyceollins are a set of soybean-specific pterocarpans with a described set of human health benefits (Pham T. H. et al., 2019). They have an anti-estrogenic effect that competes with endogenous estrogens, and are being tested as a suppressor of breast and ovarian tumorigenesis (Yamamoto et al., 2018). Glyceollin presents the distinctive feature of a fifth prenylated ring linked to the A ring (isoflavonoid core). The first steps from daidzein involve the already discussed I2′H and IFR enzymes to produce 2-hydroxy-2, 3-dihydrodaidzein. From there the enzyme PTS generates the fourth ring to obtain the molecule 3, 9-dihydroterocarp-6a-en, also reported as glycinol (Fischer et al., 1990). Subsequently, a set of prenyltransferases (PTs) convert glycinol into a whole set of precursors, where the prenylated site determines the glyceollin type (I, II or III) (Sukumaran et al., 2018). The last step is the cyclization of the fifth ring by glyceollin synthase, creating up to six different glyceollins depending on their prenyl position (Welle and Grisebach, 1988).

### 3.2 Production of coumestans

Coumestans is another important subgroup of isoflavonoid derivatives. They are usually reported as a separate group, although some authors refer to them as oxidized products of the pterocarpanoid subfamily (Goel et al., 2013). They are characterized by the addition of a keto group at position C-6, and a double bond between positions 6a and 11a. To date, more than 120 coumestans have been reported with potential clinical effects (Tu et al., 2021). The most studied compound within this family is coumestrol, which is present in soy leaves. Intake of coumestrol is associated with reduced risk for breast cancer, skin photoaging protection, and neuroprotection (Hedelin et al., 2008; Castro et al., 2014; Park et al., 2015). Coumestrol biosynthesis starts from daidzein where the previously mentioned I2′H enzyme adds a hydroxyl group to produce of 2′hydroxydaidzein. The next metabolic step is the reduction of the double bond between C-2 and C-3 (ring C) by the action of the IFR enzyme. The product obtained, 2-hydroxy-2, 3-dihydrodaidzein, is then converted into 3, 9-dihydroterocarp-6a-en by at least two unknown catalytic reactions. A combination of the VR enzyme plus the effect of the isoflav-3-ene synthase (I3S) are predicted to generate the isoflav-3-ene precursor for the characteristic fourth ring formation (Uchida et al., 2020). Finally, another two to three reactions are needed to add the keto group to C-2 of the isoflavonoid core, the distinctive feature of the coumestans subgroup. A transcriptomic study identified that up to 14 genes are associated for coumestrol biosynthesis from the precursor daidzein (Ha et al., 2019). The full metabolic pathway for coumestrol remains incomplete despite efforts to identify the genes involved on its synthesis.

Apart from coumestrol, other relevant coumestans with potential applications for human health are wedelolactone, psoralidin and glycyrol, although their biosynethic pathways are incomplete. Wedelolactone, first reported in the plant *Wedelia calendulacea*, is a coumestan skeleton with hydroxyl groups at positions 1, 8, and 9; and a methoxyl group at position 3. This metabolite has been studied for multiple clinical applications, such as anti-inflammation, anti-oxidative, inhibits breast cancer, and neuroprotective against Parkinsonism (Hsieh et al., 2015; Zhu et al., 2019; Sharma et al., 2021). Psoralidin, a coumestan first isolated from the legume *Psoralea corylifolia,* has the chemical characteristic of two hydroxyl groups at positions 3 and 9 and a prenyl group at position 2. Multiple pre-clinical studies have demonstrated its anticancer, antiosteoporotic, anti-inflammatory, anti-vitiligo, antibacterial, antiviral, and antidepressant-like effects (Sharifi-Rad et al., 2020). Lastly, glycyrol, a coumestan isolated from the Leguminosae species *Glycyrrhiza* sp.*,* presents a distinctive feature of an O-methylation at position C-1 and a prenylation at position C-2. Glycyrol demonstrates multiple clinical uses as anti-cancer, anti-inflammatory, hepatoprotective, antimicrobial, and anti-viral agents (Tu et al., 2021). Overall, several pterocarpans and coumestans are described with clinical effects; their metabolic pathway is fully reported for some compounds (medicarpin, glyceollin), and is incompletely understood for others (coumestrol, maackiain). The identification of the missing enzymes, as well as the identification of the best production systems, are the first steps for boosting the biosynthesis of pterocarpans and coumestans.

## 4 Bioprospecting strategies

### 4.1 Exploring biodiversity

Microbial bioprospecting is a new terminology, but humans have used microbes for their benefit for many years, from the implementation of yeast for bread-making to the discovery of new molecules to treat diseases. The recent emergence of metagenomics analysis has unleashed the potential of microbial bioprospecting. It is estimated that there are more than 1 × 10^16^ microbes in just 1 ton of soil, of which 85%–99% are unculturable (Curtis and Sloan, 2005; Lok, 2015). Analysis of this massive data set led to the discovery of several new strains for both human (therapeutic, food production) and environmental (sustainable industries, bioremediation) applications.

Different approaches for microbial *in silico* bioprospecting are being already applied using bioinformatic resources. The two main approaches for the identification of microbial diversity are mining data from publicly available databases, or using raw data from different sampling sites following metagenomic pipelines (Vuong et al., 2022b). Within the available datasets, online trustworthy repositories are associated with the International Nucleotide Sequence Database: the DNA Databank of Japan (DDBJ; http://www.ddbj.nig.ac.jp/), the European Molecular Biology Laboratory’s European Bioinformatics Institute (EMBL-EBI; http://www.ebi.ac.uk/ena/) the National Center for Biotechnology Information (NCBI; https://www.ncbi.nlm.nih.gov/), and the Joint Genome Institute (JGI; https://img.jgi.doe.gov). For environmental metagenomic data, global sequencing initiatives such as Tara Oceans (http://oceanmicrobiome.embl.de/), the Earth Microbiome Project (https://earthmicrobiome.org/), and the Malaspina Gene Database are excellent sources of publicly available information (Acinas et al., 2021). Multiple tools are available for exploring metagenomic data to identify protein sequences and rebuild metabolic pathways. Bioinformatic tools such as QIIME (http://qiime.org/) allow the analysis of metagenomic raw data, and once individual genomes are segregated, tools such as Prodigal (https://github.com/hyattpd/Prodigal) predict genes and link them with metabolic pathways.

Different environments have been explored for novel microbes and metabolic pathways following a metagenomic approach (Quince et al., 2017). Marine samples are a rich source of new microbes, that are bio-prospected as a source of novel genes (Paoli et al., 2022). Extreme environments from polar to volcanic regions, provide a source of extremophiles that are investigated for human health applications using a multi-omics approach (Hedlund et al., 2014). Additionally, not only natural environments are considered a rich source of microorganisms. For instance, industrial effluents are targeted for microbes that degrade lipids, a key activity for biotechnological applications (Peil et al., 2016). The enzyme lipase is then recovered using liquid biphasic flotation, efficiently and sustainably (Sankaran et al., 2018). Overall, microbial bioprospecting generates a rich source of microorganisms for a diverse range of applications, including isoflavonoids production. The following section gives an example of how microalgal species can be targeted to produce isoflavonoid derivatives.

### 4.2 Employing microalgae

Microbes are an abundant source of new nutraceutical and pharmaceutical products. As phototrophic microbes (prokaryotic cyanobacteria and eukaryotic microalgae) share biosynthetic pathways with plants, scientists are exploring these organisms as chassis to produce plant metabolites. Microalgae are photosynthetic unicellular or colonial microorganisms with the ability to grow in different soil and underwater environments. Phototrophic microbes are divided into two prokaryotic divisions: Cyanophyta and Prochlorophyta, and nine eukaryotic divisions: Glaucophyta, Rhodophyta, Heterokontophyta, Haptophyta, Cryptophyta, Dinophyta, Euglenophyta, Chlorarachniophyta and Chlorophyta (Hemaiswarya et al., 2013). Decades ago, the species *Dunaeliella salina* was successfully implemented as a novel sustainable production system for β-carotene as it naturally produces up to 10% of its dry weight as β-carotene (Harvey and Ben-Amotz, 2020). Since then, microalgal species are bio-prospected for farming (aquaculture), biomanufacturing (nutraceutical, pharmaceutical, functional foods, biofuels), and environmental (bioremediation) applications (Choong et al., 2016; Mobin and Alam, 2017).

Using microalgal species as a sustainable production chassis to produce plant metabolites present several benefits. As a 3rd generation biorefinery, microalgae utilize renewable energy (sunlight) and CO_2_ and hence microalgae are a key global contributor to CO_2_ sequestration (Liu Z. et al., 2020; Prasad et al., 2021). Some microalgal species also have the potential to use wastewater as a source of nutrients, adding another positive impact on the environment as a remediation tool (Goswami et al., 2022). Additionally, similar to the yeast *S. cerevisiae,* some microalgal species are Generally Recognized As Safe (GRAS), so they can be consumed as food (Villarruel-Lopez et al., 2017; Wells et al., 2017; Caporgno and Mathys, 2018; Khemiri et al., 2020). Even though, conducting a food safety risk assessment is imperative to ensure a high-quality product for human consumption (Wu et al., 2022).

Isoflavonoids derivatives and their associated enzymes were recently identified in algal species (Del Mondo et al., 2022). A summary of these findings is represented in Table 1. Microalgal species share similar metabolic pathways with plants probably due to a primordial ancestor, creating an exciting avenue as isoflavonoid sustainable producers (Klejdus et al., 2010; Del Mondo et al., 2021). The isoflavonoids genistein and daidzein were found in the prokaryotic Cyanobacteria *Nostoc,* as well as in eukaryotes divisions such as Chlorophyta and Rhodophyta (Goiris et al., 2014; Del Mondo et al., 2021; Ferdous and Balia Yusof, 2021). Massive microalgal blooms may have a negative impact on the environment and human health, but those species can be explored in confined laboratory conditions to source valuable genes, enzymes, and molecules. The IFS, a key protein for the biosynthesis of isoflavonoids have been found in 45 of the 47 algal taxa analyzed according to sequence alignments (BLASTp) (Del Mondo et al., 2022). The IFS plus other 28 phenylpropanoid core enzymes were screened using a multistep *in silico* analysis, that demonstrates the presence of isoflavonoid-related genes across microalgal databases (Del Mondo et al., 2022).

**TABLE 1:** Microalgal species that naturally produce isoflavonoid derivatives.

Flavonoids	Microalgal species	Environment	Concentration	References
Total flavonoid content	Cyanobacteria *Chroococcidiopsis thermalis*	Extreme environments: deserts and hot springs	2.44 mg/g dry biomass	Ijaz and Hasnain (2016)
	Cyanobacteria *Leptolyngbya* sp.	Tunisian hot springs	34.9 mg/g dry biomass	Trabelsi et al. (2016)
	Chlorophyta *Desmodesmus* sp.	Wastewater treatment system	4.03 mg/g dry biomass	Safafar et al. (2015)
	Chlorophyta *Dunaliella salina*	Hypersaline environments (salt lakes)	3.61 mg/g dry biomass	Safafar et al. (2015)
	Euglenoida *Euglena tuba*	Indian aquatic bodies	1.01 mg/g dry biomass	Chaudhuri et al. (2014)
	Chlorophyta *Chlorella sorokiniana*	Freshwater (able to grow in wastewater)	2.41 mg/g dry biomass	Safafar et al. (2015)
	Chlorophyta *Chlorella vulgaris*	Freshwater (able to grow in wastewater)	118 μg/mL culture	Yadavalli et al. (2022)
Isoflavonoid precursors				
p-Coumaric acid	Diatom *Phaeodactylum tricornutum*	Marine artic polar	750 ng/g dry biomass	Goiris et al. (2014)
	Rhodophyta *Porphyridium purpureum*	Marine environment	770 ng/g dry biomass	Goiris et al. (2014)
	Chlorophyta *Haematococcus pluvialis*	Freshwater	640 ng/g dry biomass	Goiris et al. (2014)
Naringenin	Chlorophyta *Haematococcus pluvialis*	Freshwater	0.6 ng/g dry biomass	Goiris et al. (2014)
	Haptophyta *Diacronema lutheri*	Marine environment	0.6 ng/g dry biomass	Goiris et al. (2014)
	Cyanobacteria *Leptolyngbya* sp.	Tunisian hot springs	4.1 ng/g dry biomass	Trabelsi et al. (2016)
Isoflavonoids				
Daidzein	Diatom *Phaeodactylum tricornutum*	Marine artic polar	5.9 ng/g dry biomass	Goiris et al. (2014)
	Rhodophyta *Porphyridium purpureum*	Marine environment	1.27 ng/g dry biomass	Goiris et al. (2014)
	Chlorophyta *Haematococcus pluvialis*	Freshwater	0.6 ng/g dry biomass	Goiris et al. (2014)
	Cyanobacteria *Nostoc 17*	Marine, freshwater and terrestrial	7.05 ng/g dry biomass	Klejdus et al. (2010)
	Chlorophyta *Scenedesmus* sp.	Marine, freshwater and terrestrial	10.59 ng/g dry biomass	Klejdus et al. (2010)
Genistein	Diatom *Phaeodactylum tricornutum*	Marine artic polar	1.42 ng/g dry biomass	Goiris et al. (2014)
	Rhodophyta *Porphyridium purpureum*	Marine environment	0.63 ng/g dry biomass	Goiris et al. (2014)
	Chlorophyta *Haematococcus pluvialis*	Freshwater	0.4 ng/g dry biomass	Goiris et al. (2014)
	Cyanobacteria *Nostoc 17*	Marine, freshwater and terrestrial	5.91 ng/g dry biomass	Klejdus et al. (2010)
	Chlorophyta *Spongiochloris spongiosa*	Freshwater and terrestrial	4.27 ng/g dry biomass	Klejdus et al. (2010)
	Chlorophyta *Scenedesmus* sp.	Marine, freshwater and terrestrial	6.11 ng/g dry biomass	Klejdus et al. (2010)
Formononetin	Cyanobacteria *Nostoc 17*	Marine, freshwater and terrestrial	33.14 ng/g dry biomass	Klejdus et al. (2010)
	Chlorophyta *Spongiochloris spongiosa*	Freshwater and terrestrial	4.29 ng/g dry biomass	Klejdus et al. (2010)
	Chlorophyta *Scenedesmus* sp.	Marine, freshwater and terrestrial	5.92 ng/g dry biomass	Klejdus et al. (2010)
Coumestrol	Cyanobacterial multispecies blooms	Czech lakes and ponds	0.1 ng/L water sampled	Procházková et al. (2017)
IFS Enzyme	45 different algae taxa	Multiple environments	NA	Del Mondo et al. (2022)

Besides the identification of microalgal species that can naturally produce isoflavonoids, strategies should be followed to boost metabolite levels to generate a commercially viable solution. In recent years, engineering tools have been developed for some phototrophic microorganisms such as cyanobacteria, chlorophytes, diatoms, and eustigmatophytes. For these organisms, culture conditions and genetic edition tools were optimized (Vavitsas et al., 2021). Phototrophic unicellular microorganisms have already shown immense potential as sustainable production platforms, and the yet unexplored field of microalgal isoflavonoid production may soon become a reality.

### 4.3 Optimizing metabolic pathways

**FIGURE 2:**
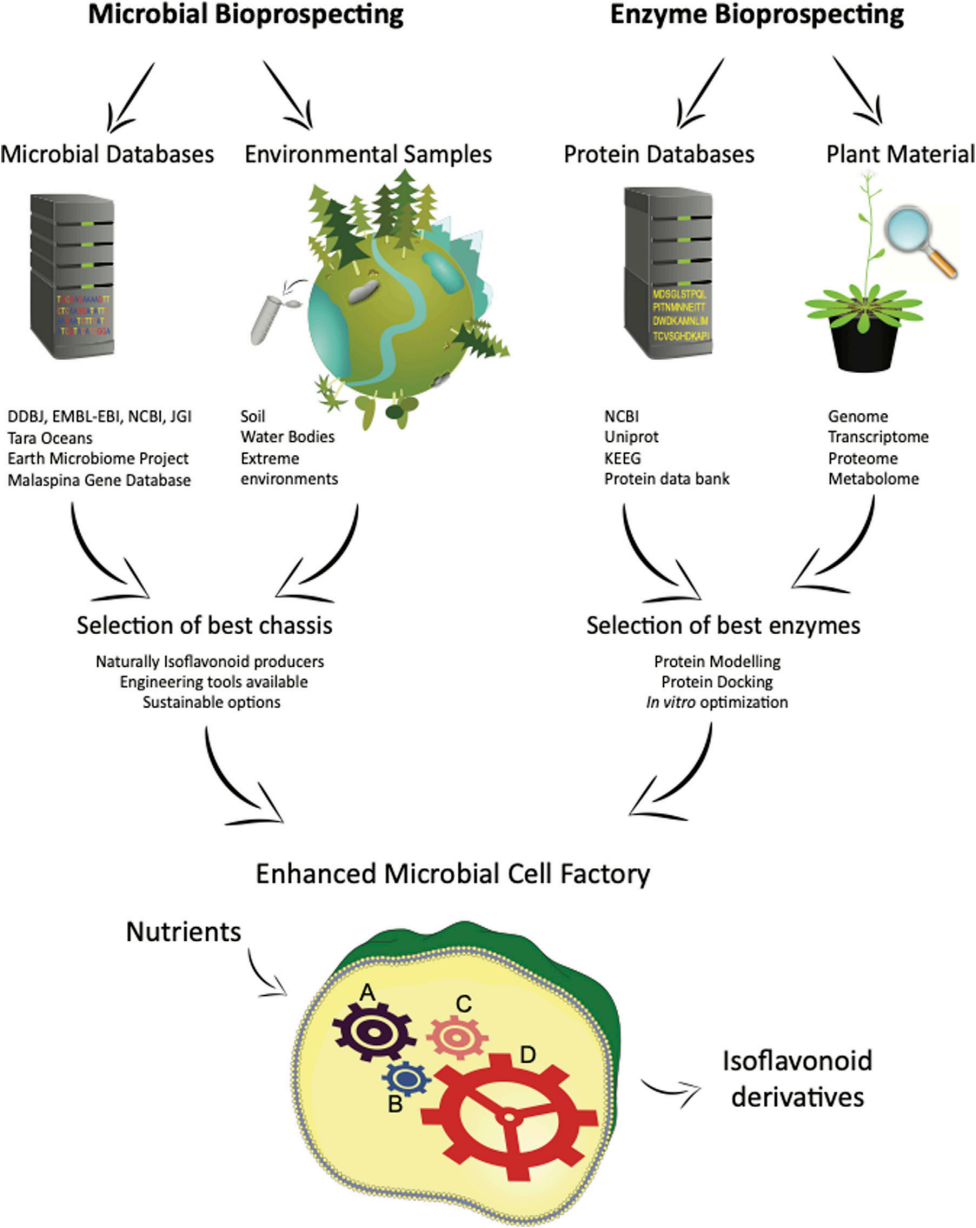
Bioprospecting strategies for microbial and enzyme bioprospecting. Microbial bioprospecting allows the identification of best chassis, whereas enzyme bioprospecting identifies the best enzymes for the production of isoflavonoid derivatives using microbial cell factories.

Enzymes with enhanced activity is a particularly effective strategy for boosting metabolite production. The approach is slightly different to microbial bioprospecting, where instead of using metagenomic data from the environment, an organism’s protein database (proteomics) is combined with its metabolic expression for discovering and optimizing enzymatic reactions. A diagram representing both microbial and enzyme bioprospecting strategies is shown in Figure 2.

The identification of the state-of-the-art is the first step for the discovery of enzymes and whole metabolic pathways. As multiple steps are required for the discovery of the best enzymes, a summary of enzyme bioprospecting tools divided by stages is detailed in Table 2. To identify biosynthetic gaps, the presence of metabolites and their precursors can provide information about enzymatic reactions. Metabolite detection using High-Performance Liquid Chromatography (HPLC) or Mass Spectrometry (MS) analysis evaluates chemo-structural diversity to build enzymatic pathways (Santana et al., 2021). Nuclear magnetic resonance (NMR) spectroscopy is another approach that allows the identification of potentially bioactive compounds using the whole plant metabolomic set (Augustijn et al., 2021). The biosynthetic pathway of the enzymes involved in the generation of those metabolites can be predicted using retro-analysis tools, linking the precursors with the final product (Delépine et al., 2018). A machine learning approach allows not only to predict one metabolic pathway but the whole organism’s metabolomics, using genomic, transcriptomic and proteomic information altogether (Zampieri et al., 2019). A different approach is followed when the metabolic information is already known. The database KEGG is the most cited source of already fully described metabolic pathways and compound relationships. Multiple public protein databases provide information about proteins, such as Joint Genome Institute (JGI; https://jgi.doe.gov/), Uniprot (https://www.uniprot.org) and Protein Data Bank (PDB; https://www.rcsb.org/). The NCBI database, as well as Uniprot, allow the short-listing of homologous proteins as potential candidates for biosynthetic pathways.

**TABLE 2:** Online sources and tools for enzyme bioprospecting.

Enzyme bioprospecting sources and tools	Application	References
Protein databases (source of information)		
Joint genome institute	Multispecies DNA, RNA and protein database (annotations and distribution)	Grigoriev et al. (2012)
NCBI	Multispecies DNA, RNA and protein database (annotations, distribution, blast)	Jenuth (1999)
Uniprot	Find, align and blast proteins	The UniProt Consortium (2019)
KEGG	Exploration of metabolic pathways and enzymes involved	Kanehisa and Goto (2000)
Protein data bank (PDB)	Experimentally determined 3D structures and computed protein models	Dutta and Berman (2005)
Pathway and enzyme discovery		
Retro-analysis	Discover biosynthetic pathways using metabolite’s information	Delépine et al. (2018)
Metabolic modelling	Discover biosynthetic pathways using information from multiple omics	Zampieri et al. (2019)
Enzyme characteristics (Protein Modelling)		
ProtParam (ExPaSy resource portal)	Calculate protein main parameters	Artimo et al. (2012)
SWISS-MODEL	Protein modelling	Waterhouse et al. (2018)
ALPHAFOLD	Protein Structure Database and modelling	Jumper et al. (2021)
ColabFold	Protein modelling combining homology search technologies	Mirdita et al. (2022)
MEME Suite Tools	Protein motif discovery and analysis	Bailey et al. (2015)
Enzyme-ligand interaction (protein docking)		
AutoDock and Vina	Protein-ligand affinity interaction (PyMOL visualization)	Seeliger and de Groot (2010)
CB-Dock	User friendly web-based for protein-ligand affinity	Liu et al. (2019)
HADDOCK	Improved protein-ligand docking considering homology conformations	Koukos et al. (2021)
DEELIG	Deep-learning-based method for protein-ligand determination	Ahmed et al. (2021)
Protein Docking of Interfacial Enzymes	Virtual screening that considers both catalytic and membrane-interaction domains	Schaller et al. (2022)
*In vitro* techniques for further optimization		
directed evolution, random mutagenesis, error-prone amplification	Generation of unnatural homology variants	Kamble et al. (2019)
Enzyme scaffold substrate channeling	Improve biosynthetic cascades linking enzymes together	Ellis et al. (2019)

After identifying the target enzyme within a specific biosynthetic pathway, the first step for boosting its activity is to model its 3D structure. Protein modelling is performed using tools such as ProtParam software (https://web.expasy.org/protparam/), SWISS-MODEL (https://swissmodel.expasy.org/) and Alphafold (https://alphafold.ebi.ac.uk/) to predict the protein conformation, kinetic parameters and physicochemical properties (extinction coefficient, estimated half-life, instability index, and aliphatic index). The search is usually based on full protein homology and domain structures, and motif analysis tools such as the MEME Suite Tool can provide an extra layer of enzymatic information (Bailey et al., 2015). Molecular docking is the next step that allows an *in silico* test of protein-ligand interactions. This virtual screening estimates the ligand position within the modeled protein cavity and the potential amino acids involved in the enzymatic reaction. Several tools are described in the literature for applying this technology to discover novel enzymes with improved activity, considering rigid and flexible dockings (Fan et al., 2019). Within these tools, the popular molecule-viewing software PyMOL is widely used with the docking suite AutoDock and Vina (Seeliger and de Groot, 2010). A rectangular box defines the binding ligand-protein site, from where multiple binding runs are performed between the ligand and the specific amino acids, to determine the binding energies. Cutting-edge techniques like High Ambiguity Driven DOCKing (HADDOCK) incorporate the factor of ligand conformation, adding an additional layer of information in the dynamic landscape of this field (Koukos et al., 2021). There are also user-friendly and web-based tools, such as CB-Dock, that retain the power of protein docking (Autodck Vina) without compromising the quality of the results (Liu Y. et al., 2020). In the last few years, new approaches have been developed using the power of deep learning and convolutional neural networks to “train” the model for the characterization of multiple protein-ligand affinity interactions. As an example, the deep-learning-based approach (DEELIG) uses this method (Ahmed et al., 2021). For interfacial enzymes, the protein motifs (catalytic and membrane binding) are analyzed separately, to build homology models and docking activity data (Schaller et al., 2022). The implementation of all these strategies allows researchers to predict protein-ligand binding affinity and overall enzymatic activity to select the more suitable enzymes for a target reaction.

Presently, *in silico* enzyme bioprospecting is positively impacting the way where novel enzymes are screened, optimized, and tested, in a cost-effective manner. Nevertheless, an *in vitro* approach is necessary to confirm the enzyme’s pharmacokinetic and predicted activity. Conventional enzyme engineering tools such as directed evolution, random mutagenesis, and error-prone amplification can add variability to the discovery of novel enzymes (Kamble et al., 2019). Once every enzymatic step is optimized for the best activity, multienzyme scaffolds can also contribute to improving biosynthetic cascades through substrate channeling, linking all the enzymes involved in a concise subcellular space (Ellis et al., 2019). Altogether, *in silico* and *in vitro* strategies enhance the biosynthetic pathway efficiency and positively contribute to obtain higher production titers.

## 5 Approaches for the production of pterocarpans and coumestans

Plant-based systems (homologous biosynthesis) are the main source of coumestans and pterocarpans, but have several limitations. First, plant-based systems are unsustainable due to the consumption of environmental resources (land, water, fertilizers). Second, the target plant biomass usually requires long growing periods to reach the harvest stage. Third, the concentration of coumestans and pterocarpans is low when compared with other isoflavones, and difficult to isolate and purify from the plant tissue. As an example, coumestrol from *Glycine max* cultivar “Santa rosa” (high coumestrol concentration) showed 1.85 μg/g dry material, while the same cultivar contains 560 μg/g dry material of the precursor daidzein (Mazur et al., 1998). Some of the plant-based production limitations can be solved by plant-tissue culturing. Coumestrol biosynthesis was obtained through adventitious soybean root cultivation, but it is still an unsustainable and cost-ineffective solution (Lee et al., 2022). Another production strategy is chemical synthesis, where the metabolite can be solely synthesized using chemical approaches. This process usually uses hazardous and expensive reagents, as is the case for the full chemical production of medicarpin (Yang et al., 2017). To some extent, a chemical reaction can be used to further modify plant metabolites, adding methyl or glycosyl groups to enhance bioavailability. This has been achieved for flavonoid production, where bioavailability, anti-inflammatory, and anticancer activities were enhanced by chemically adding methyl groups (Wen et al., 2017).

### 5.1 Evaluating microbial hosts

The application of microbial-based production systems (heterologous biosynthesis), empowered by bioprospecting, may provide an alternative solution for overcoming the limitations previously described by plant-based and chemical approaches. This lab-based production method does not require arable land, is scalable, sustainable, and cost-effective depending on the chassis (microbe) utilized. Granted, there are challenges that the utilization of microbial-based systems for plant natural products still need to overcome. The access distribution to this technology is mainly dominated by the United States, and the patenting of plant biosynthetic pathways may threaten small-acre producers from developing countries (French, 2019). A global regulatory framework is essential to guarantee fair access to this technology, and to protect the original sources of natural products.

The chassis selection plays a central role in the production of plant metabolites. The commonly used model species, *Escherichia coli* and *S. cerevisiae,* are largely employed as there are numerous of engineering tools available (Cravens et al., 2019). Between these two organisms*, S. cerevisiae* is more suitable for plant metabolites as it naturally contains the plant machinery (Cytochrome P450 enzymes and others) required for plant metabolite production. *S. cerevisiae* is also Generally Recognized As Safe (GRAS), being an excellent candidate to produce food supplements and other nutraceutical derivatives (Singh and Gaur, 2021). Other GRAS yeast species such as *Aspergillus sp*. and *Hansenula polymorpha* have been used for the expression of plant products (Pham J. V. et al., 2019). Bioprospecting strategies have already been implemented to identify bacterial host systems compatible for large-scale cultivation. Poorly investigated microorganisms such as the Gram-positive *Streptomyces sp.*, *Bacillus sp.*, and *Lactococcus lactis* are also promising cell factories for the production of produce plant chemicals. Different microbial production strategies have been explored for the production of isoflavonoids (Chouhan et al., 2017; Sajid et al., 2021b). Table 3 summarizes the current reports for coumestans, pterocaptans precursors and other isoflavonoid derivatives. The microbial-based production of the precursors p-coumaric acid, liquiritigenin and daidzein have already been achieved using both bacterial and yeast species, but the biosynthesis of coumestans and pterocarpans is incompletely described in the literature. In the case of coumestans (coumestrol), a heterologous production is still not feasible as its biosynthetic pathway is not fully described. Similarly, the *de novo* biosynthesis of medicarpin is complex and difficult to optimize as it requires several enzymatic reactions.

**TABLE 3:** Successful examples of heterologous biosynthesis of isoflavonoid precursors using different cell factories. Abbreviations: ARO10, phenylpyruvate decarboxylase; PDC5, pyruvate decarboxylase; TAL, tyrosine ammonia-lyase; ARO4, 3-deoxy-D-arabino-heptulosonic acid synthase; ARO7, chorismate mutase; AROL, shikimate kinase II; 4CL, 4-coumaroylCoA ligase; CHS, chalcone synthase; CHI, chalcone isomerase; CHR, chalcone reductase; IFS, isoflavone synthase; HIS, 2-hydroxyisoflavanone synthase; HID, 2-hydroxyisoflavanone dehydratase; PAL, phenylalanine ammonia lyase; C4H, cinnamate 4-hydroxylase; ACC, acetyl-CoA carboxylase; CCL, cinnamate/coumarate-CoA ligase; CPR, cytochrome P450 reductase.

Precursors	Microbial cell factory	Substrate	Titer	Heterologous enzymes and other strain modifications	Strategy	References
Pterocarpans and coumestans precursors						
p-Coumaric acid	*E. coli*	Tyrosine	2.5 gr/L	TAL	Screening and overexpression of multiple TAL variants	Jones et al. (2017)
	*S. cerevisiae*	Tyrosine	1.9 gr/L	TAL, ARO10 and PDC5 (knock-out), ARO4, ARO7 and AROL (overexpression)	Reduction of by-products and overexpression of precursors	Rodriguez et al. (2015)
Liquiritigenin	*E. coli*	p-Coumaric acid	7.6 mg/L	4CL2, CHR, CHS, CHI	Different copy number plasmids to control enzyme expression	Yan et al. (2007)
	*S. cerevisiae*	Tyrosine	5.3 mg/L	TAL, 4CL, CHR, CHS, CHI	p-Coumaric acid overexpression yeast plus liquiritigenin metabolic pathway	Rodriguez et al. (2017)
	*Yarrowia lipolytica*	Phenylalanine	62.4 mg/L	PAL, 4CL, CHS-CHR (fused), CHI	Strain engineering adding 4 genes plus 1 fusion enzyme	Akram et al. (2021)
Daidzein (isoflavonoid)	*E. coli*	Liquiritigenin	18 mg/g dry weight	IFS	IFS engineering for using P450 enzymes in bacteria	Leonard and Koffas (2007)
	*S. cerevisiae*	Glucose	85.4 mg/L	PAL, C4H-4CL (fused), CHS, CHR-CHI (fused), HIS, HID	Selection of best homologous candidates, control expression with promoters, precursors overexpression and protein fusion	Liu et al. (2021)
Other Isoflavonoids precursors						
Naringenin						
	*E. coli*	Tyrosine	57 mg/L	PAL (TAL activity), C4H, 4CL, CHS, CHI, ACC (overproduce manolyl-CoA), ScCCL (overproduce 4-coumaroyl-CoA)	Metabolic strain engineering for overexpressing precursors	Miyahisa et al. (2005)
	*S. cerevisiae/E. coli*	D-xilose	21.1 mg/L	TAL, CHS, 4CL, CHS, CHI (*S. cerevisiae*); pyk and pheA gene knock-out (*E. coli*) to overexpress acetate and tyrosine	Co-culture with multiple copies of 4CL into an extra plasmid	Zhang et al. (2017)
	*S. cerevisiae*	Tyrosine	90 mg/L	TAL, CHS, 4CL, CHS, CHI	Engineered strains to overexpress precursors tyrosine and malonyl-CoA	Lyu et al. (2017)
*Genistein (isovlavonoid)*	*E. coli*	p-Coumaric acid	18.6 mg/L	4CL, CHS, IFS, CPR	Inclusion of P450 machinery in bacteria and co-culture for split the pathway	Kim (2020)
	*S. cerevisiae/E. coli*	Tyrosine	6 mg/L	PAL, 4CL, CHS, CHI (*E. coli*), IFS (*S. cerevisiae*)	Co-culture for achieving higher titer expressing P450 enzyme IFS in yeast	Katsuyama et al. (2007)
	*S. cerevisiae*	Phenylalanine	0.1 mg/L	PAL, C4H, 4CL, IFS, CPR, CHS, CHI	Introduction of a *de novo* biosynthetic pathway in one strain	Trantas et al. (2009)

A “divide and conquer” strategy may ease the metabolic burden of incorporating multiple genes in one strain. This co-culture strategy also allows the exploration of branched molecules combining different enzyme cascades. The selected metabolic pathway is separated and integrated into different strains, which can be the same or different species (Chen et al., 2019). As an example, an *E. coli* bi-culture was used to produce the flavonoid catechin using one strain to obtain naringenin (upstream precursor) while the second strain uses the precursor to generate the target metabolite (Jones et al., 2016). For isoflavonoids, up to 6 mg/L of the metabolite genistein was produced by dividing the pathway between bacterial and yeast species (Katsuyama et al., 2007). The use of polycultures is an efficient strategy for the biosynthesis of complex molecules. For instance, multiple enzymatic reactions were divided into several strains of *E. coli* to produce anthocyanins from simple sugars (Jones et al., 2017). Also, a symbiotic relationship can be established by forming a consortium where one species provides substrates to the other one and *vice versa.* This is the case of co-culturing *S. cerevisiae* with microalgal species, in which the algae produce essential nutrients and the yeast provides extra CO_2_ (Nguyen et al., 2020; Alam et al., 2022). Overall, the use of bioprospecting strategies for sourcing better enzymes and microbial chassis offers a promising strategy for boosting plant metabolite production.

Apart from the model species, *E. coli* and *S. Cerevisiae*, other microbes need be explored as production chassis for the production of pterocarpans and coumastans. The presence of pterocarpans and coumastans in cyanobacterial blooms indicates that the full metabolic pathway is already present in phototrophic bacteria (Procházková et al., 2017). Daidzein, a precursor of pterocarpans and coumastans, was found both in prokaryotic and eukaryotic microalgal species (Klejdus et al., 2010; Goiris et al., 2014). The addition of three to four enzymatic steps will allow the production of pterocarpans and coumastans from those microalgal species. Furthermore, the activation of promoters through nuclease-dead Cas9 systems can help to overexpress key enzymes of the pathway, as was demonstrated in rice plants (Gong et al., 2020). Lastly, the use of co-culture between yeast and microalgal species presents a strategy for enhancing secondary metabolite production, not yet been explored for pterocarpans and coumestans. Scientific advances in recent years provide an excellent set of tools to unleash the production of pterocarpans and coumastans. Filling the enzyme characterization gaps, and selecting the best production chassis would help to meet the escalating demand for these isoflavonoid derivatives.

## 6 Evaluating enzyme orthologs

Key enzymes for pterocarpans and coumenstans production such as OMT, I2H, IFR, VR, and PTS were originally identified from enzyme characterization studies. The OMT enzyme, key for the conversion of daidzein to formononetin, was first characterized in the legumes *Medicago truncatula* and *Glycyrrhiza echinate* (Akashi et al., 2003; Liu et al., 2006; Li et al., 2016)*.* More recently, another enzyme from the species *Pueraria lobata* (PIOMT9) was identified as an alternative route for formononetin biosynthesis (Li et al., 2016). The membrane-bound CYP450 protein I2′H, key to hydroxylate isoflavones, was cloned and expressed in *E. coli* to test its ability to act on formononetin. The subtype sequence from *Lotus japonicus* generated 8.4 mg/L of hydroxyformononetin, while the *G. echinate* variety failed to produce that compound (Uchida et al., 2015). An ortholog from *Astragalus membranaceus* was also cloned and purified, but its functional activity was not fully reported (Chen et al., 2015). The IFR enzyme from *G. max* was purified and characterized to detect its activity *in vitro,* and overexpressed in soybean seeds to confirm the overproduction of glyceollins (Cheng et al., 2015). The IFR subtype from *Medicago sativa* was successfully implemented to produce vestitone (Uchida et al., 2017). The VR orthologous sequence from *M. sativa* was tested using an *E. coli* chassis, and its activity was tested to produce DMI (Guo and Paiva, 1995). The activity of PTS, a key enzyme for pterocarpan biosynthesis, was characterized for *Glycyrrhiza echinata*, *L. japonicus*, and *G. max* species. From that study, the *G. echinate* ortholog was identified as the most efficient for medicarpin production (Uchida et al., 2017).

Enzyme characterization using *in silico* tools is a useful approach for enhancing the heterologous production of pterocarpans and coumenstans. Flavones and flavonol synthases have been bio-prospected using some of the tools previously described. In an interesting approach, researchers curated 44 enzymes using PyMOL and AutoDock Vina for protein docking and based on the instability index and conserved domain data selected the best candidates (Wang et al., 2021). In a recent publication, the IFS, a critical enzyme for the biosynthesis of isoflavonoids, was analyzed by considering its interaction with the molecules, liquiritigenin and naringenin. The use of Alphafold and Swiss-Model for the modelling, and AutoDock tools for the docking, allowed the authors to determine *in silico* the IFS from *Trifolium pretense* as the best candidate for liquiritigenin conversion (Sajid et al., 2022). For pterocarpans and coumestans production, the enzyme VR has previously been crystallized, modelled and docked *in silico* to test its ability to produce DMI (Shao et al., 2007). The enzyme PTS, key in the cycling stage for producing the fourth ring structure, was recently analyzed using protein docking with the ligand DMI. The PTS enzyme from licorice (*G. echinata*) was determined as the best dirigent protein for the formation of both (−) and (+)-medicarpins (Meng et al., 2020). Besides these analyses, no other efforts have been reported so far for modelling and docking enzymes related to pterocarpans and coumestans biosynthesis.

## 7 Challenges and prospects

The main challenge that the scientific community will face shortly is how to apply sustainable production systems to cope with the increasing global demand for isoflavonoids. The discovery of a novel production chassis through microbial bioprospecting may help to identify more efficient and sustainable solutions. Bioprospecting allows the discovery of novel microorganisms that produce isoflavonoid derivatives, besides plant species. Phototropic microbes such as chlorophyta and cyanobacteria may become an alternative chassis, boosting metabolite production, and conserving the environment at the same time. Notably, the use of these species are potentially beneficial to the environment on account of their capacity to act as carbon-sinks and wastewater remediators, in line with the United Nations Sustainable Developments Goals (Vuong et al., 2022a). Efforts should be implemented to develop more engineering tools for algal species. A genetic toolkit is available for diatoms and cyanobacteria species, but gene delivery, transformation, and selection of recombinant microalgal strains require significant improvement to replace model species *S. saccharomyces* and *E. Coli* (Lee et al., 2023).

A full comprehension of the metabolic pathway is essential to determine the enzymes involved in pterocarpans and coumestans biosynthesis. Enzyme bioprospecting allows the identification and selection of the best enzyme homologs, using *in silico* bioinformatic tools for modelling and docking simulations. Although some pterocarpans and coumestans metabolic enzymes were analyzed, such as the IFS and PTS, further contributions should deliver more information about the best homologous enzymes for the whole pathway. Once the best production chassis and enzymes are identified, microbial-based systems should be implemented to boost pterocarpans and coumestans expression. The employment of a symbiotic poly-culture strategy may positively contribute to the target molecule production, and ease the optimization of fermentation conditions. Other tools that can help to boost production titer besides the incorporation of the metabolic pathway are: a) determining the perfect expression ratio of all the enzymes involved through techniques such as multiplex automatic genome engineering (MAGE) (Wang et al., 2009), b) implementation of strains with an increased expression of precursors and cofactors (Akhtar and Jones, 2014), c) applying substrate channeling through enzyme scaffolds and protein fusions (Kang et al., 2018; Choi et al., 2019). Overall, a link between researchers with metabolic pathway/enzymes background, and engineering specialists who work on microbial sustainable production systems may overcome the isoflavonoid production bottleneck.

## 8 Conclusion

Isoflavonoids derivatives such as pterocarpans and coumestans have applications in multiple fields. Diseases such as breast cancer, osteoporosis and neurodegenerative syndromes can be targeted using these molecules to improve human health and wellbeing. Nevertheless, further experimental contributions are needed to enhance their production, purification and overall implementation. Though efforts have been implemented to identify the biosynthetic pathways of some pterocarpans and coumestans, others remain unclear. A holistic bioprospecting strategy that considers both pathway identification (enzymes) and microbial discovery (production systems) is very much required. We describe the “state of the art” microbial-based systems for pterocarpans and coumestans production, and propose the implementation of microalgae species as sustainable production platforms. This strategy is not limited to isoflavonoid derivatives, and can be applied to other plant natural product such as terpenes, steroids, other phenolics. The shared knowledge contributed by the scientific community regarding the bioprospecting of enzymes and microbes will be invaluable for the development of production platforms for the isoflavonoid derivatives, pterocarpans and coumestans.
